# Bistability in the actin cortex

**DOI:** 10.1186/1757-5036-3-12

**Published:** 2010-06-24

**Authors:** Carsten Beta

**Affiliations:** 1 Institut für Physik und Astronomie, Universität Potsdam, 14476 Potsdam, Germany; 2 Max-Planck-Institut für Dynamik and Selbstorganisation, 37073 Göttingen, Germany

## Abstract

Multi-color fluorescence imaging experiments of wave forming *Dictyostelium *cells have revealed that actin waves separate two domains of the cell cortex that differ in their actin structure and phosphoinositide composition. We propose a bistable model of actin dynamics to account for these experimental observation. The model is based on the simplifying assumption that the actin cytoskeleton is composed of two distinct network types, a dendritic and a bundled network. The two structurally different states that were observed in experiments correspond to the stable fixed points in the bistable regime of this model. Each fixed point is dominated by one of the two network types. The experimentally observed actin waves can be considered as trigger waves that propagate transitions between the two stable fixed points.

PACS Codes: 87.16.Ln, 87.17.Aa, 89.75.Fb

## 1. Background

The cytoskeleton is a dynamical scaffolding that determines shape and mechanical properties of a eukaryotic cell. It is composed of a cross-linked biopolymer network, in which filaments of different stiffness can be distinguished. Out of the three major classes of cytoskeletal filaments, semiflexible polymeric actin (F-actin) is mostly concentrated at the inner side of the plasma membrane. Actin filaments grow and decay in a continuous treadmilling process, so that the network structure of the actin cytoskeleton undergoes constant rapid reshaping [1]. The dynamical properties of the actin cytoskeleton play an essential role for various cellular functions including cell motility, division, and phagocytosis [2,3]. The past decade has seen a rapid advance in understanding the molecular mechanisms of actin polymerization [4]. A minimal set of essential proteins could be identified to reconstitute polymerization-driven pathogen motility *in vitro *[5]. Besides monomeric actin and ATP, such motility media typically contain a number of essential cytoskeletal components, among them the Arp2/3 complex, a central building block of dense cortical actin networks in living cells [6]. At the same time, numerous further players could be identified that control the actin machinery *in vivo *[2]. Well-known examples of such regulators are the SCAR/WAVE proteins, members of the WASp (Wiscott-Aldrich Syndrom protein) family that control the activity of the Arp2/3 complex [7].

As more and more of the molecular details of actin dynamics are elucidated, interests have recently shifted to emergent phenomena in the actin system that include a rich variety of spatiotemporal patterns. Temporal oscillations have been observed in both living and synthetic systems [8-10]. Also coherent wave patterns were found, including lateral membrane waves [11,12] and propagating waves of the Hem-1/Nap1 component of the SCAR/WAVE complex at the leading edge of human neutrophils [13]. Different theoretical approaches have been proposed that explain such observations. Time-periodic behavior was modeled by approximating the actin system as an active polar gel [14] or by considering a polymer brush model of crosslinked actin filaments close to an obstacle [15]. Such models may also include spatial degrees of freedom and yield various space-time patterns like asters, moving spots and waves [14,16].

**Figure 1:**
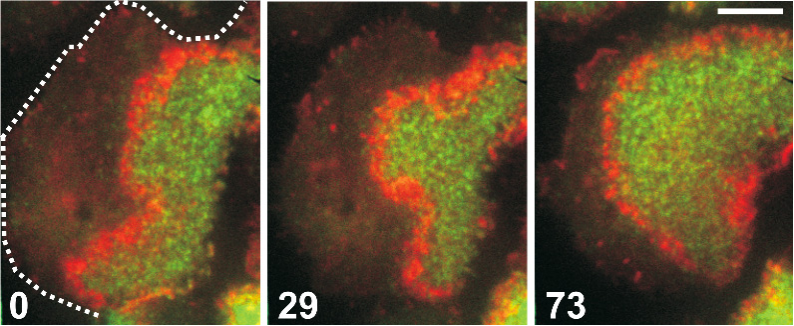
**Actin waves at the border of PIP3-rich areas**. Propagation of an actin wave (red) at the border of a PIP3-rich area (green) of the plasma membrane. A cell of *Dictyostelium discoideum *is spread on a glass surface and imaged using TIRF microscopy. During propagation of the actin wave, the cell shape remains almost unchanged; the border of the PIP3-depleted area is indicated in the first frame by a dotted line. The cell has been labeled using mRFP-LimEΔ for filamentous actin and PHcrac-GFP for PIP3. The cell is in an extensive wave-forming state during the recovery of actin polymerization from treatment with LatA. Time after the first frame is indicated in seconds. Bar 5 *μ*m. Data from [22].

The present work was motivated by studies of traveling actin waves in *Dictyostelium discoideum *cells that were performed by Günther Gerisch and co-workers. Although already reported a number of years ago [17,18], a detailed analysis of their supramolecular structure and dynamics was not performed until recently. The study of actin waves was greatly facilitated by an experimental protocol that allows to induce a phase of intense wave formation following treatment with Latrunculin A (LatA) [19]. During recovery from LatA treatment, immobile spots of actin are formed on the cell membrane. These spots become mobile and eventually give rise to traveling actin waves [19]. In a recent theoretical contribution, this transition from actin spots to waves has been successfully modeled using a FitzHugh-Nagumo-type activator-inhibitor model [20]. Note however that actin waves are also regularly observed under normal conditions [17]. The three-dimensional structure of actin waves was analyzed using spinning disc confocal microscopy, elucidating the distributions of various regulatory components and motor proteins inside the wave [21]. Fluorescent labeling of phosphoinositides revealed that actin waves separate membrane domains of high and low phospatidylinositol-(3, 4, 5) trisphosphate (PIP3) concentration [22], see Figure 1 for an example. It shows total internal reflection fluorescence (TIRF) microscopy images of a *Dictyostelium *cell that carries red and green fluorescent labels tagged to markers of filamentous actin and PIP3, respectively. It can be clearly seen that the area circumscribed by the wave exhibits high concentrations of PIP3. Note that these domains can be considered as spontaneously generated in-plane phagocytic cups. A detailed discussion of the role of actin waves in phagocytosis can be found in Ref. [22].

**Figure 2:**
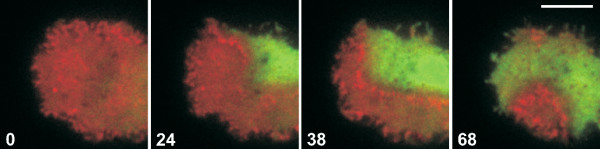
**Actin waves at the interface of different organizational states in the cell cortex**. On the two sides of a propagating actin wave, different actin structures are found. A cell of *Dictyostelium discoideum *has been labeled with mRFP-ARPC1, a subunit of the Arp2/3 complex (red), and GFP-cortexillin I (green). The Arp2/3 complex is responsible for the nucleation of branched actin filament structures, cortexillin causes actin filaments to bundle preferentially in anti-parallel direction. The cell has been imaged using TIRF microscopy. It is in an extensive wave-forming state during the recovery of actin polymerization from treatment with LatA. Time after the first frame is indicated in seconds. Bar 5 *μ*m. Data from [23].

In a recently completed work, the actin structure in wave-forming cells was explored with fluorescence microscopy [23]. Using multi-color fluorescent labeling of proteins that are associated with different arrangements of actin filaments, it was found that actin waves separate domains of structurally different actin organization. On one side of the wave, a domain enriched in the Arp2/3 complex was observed, indicating a dense dendritic texture of the actin network. This area coincided with the PIP3-rich membrane regions mentioned above. On the other side of the wave, the presence of cortexillin I and myosin II revealed a bundled arrangement of anti-parallel actin filaments. An illustration of this separation can be seen in Figure 2. TIRF microscopy images of a *Dictyostelium *cell are shown that carries a red fluorescent label on a subunit of the Arp2/3 complex and a green label tagged to cortexillin. During wave formation, a clear separation into two domains can be observed, one of them characterized by a high concentration of the Arp2/3 complex, the other by high levels of cortexillin. The actin waves are found at the boundary of these domains and may propagate with changing direction, so that the two areas reciprocally increase or decrease in size. For more details on the experimental study of actin waves the reader is referred to the contribution by Günther Gerisch in the same volume of this journal. The experimental findings suggest that in the regime of wave formation, the actin system exhibits bistable dynamics. The two structurally different states that are characterized by high concentrations of Arp2/3 or cortexillin correspond to two stable fixed points in the dynamics of the actin system. The actin waves then play the role of trigger waves that propagate transitions from one stable fixed point to the other. We use the term bistability following the conventions from the literature on pattern formation in nonequilibrium dynamical systems [24]. Slightly deviating definitions can be found in the molecular cell biology literature that are focused on rapid switching events, see *e.g. *[25] and references therein.

## 2. Results

We propose a simplified model of actin dynamics to account for the experimental observations that were reported in the previous section. In the cell cortex, F-actin may occur in a hierarchy of organizational states that range from individual actin filaments over loose meshworks to closely connected structures and thick bundles. The latter consist of a large number of densely packed filaments. Such structures are constantly formed and degraded by actin polymerization and depolymerization. As a result of this treadmilling process a continuous rearrangement of the actin cortex can be observed [1].

In the following, we will, for simplicity, assume that there are only two structurally different organizational states in the actin cortex. We will refer to these states as the bundled and the dendritic actin network. The dendritic state is characterized by a dense fabric of randomly oriented and loosely interconnected actin filaments. The Arp2/3 complex is a central building block of this network. The bundled state, on the other hand, is composed of a meshwork of thick filament bundles. Each bundle constists of several filaments that are oriented in anti-parallel fashion. Cortexillin and myosin II are associated with this network. Depending on functional requirements, one or the other network structure may locally dominate. However, under normal conditions, both structures coexist. Together, they form the actin cortex of a living cell.

Let us phrase a simple dynamical model to describe the interplay of these two network structures. The average local concentrations of the bundled and the dendritic networks are denoted with *b *and *d*, respectively. Both networks are constantly generated and degraded by actin polymerization and depolymerization. We denote the polymerization rates for the bundled and the dendritic networks with *k*_*b*1 _and *k*_*d*1_. Besides growth of existing structures by polymerization, these rates account for the nucleation of new F-actin structures. Depolymerization is taken proportional to the amount of actin network present, *i.e.*, it proceeds with rates given by *k*_*b*2_*b *and *k*_*d*2_*d*. It has been proposed that actin polymerization is regulated via a positive feeback loop that involves PIP3 and Ras [26]. Experiments with the PI3-kinase inhibitor Ly-294002 have shown that the formation of actin waves is indeed dependent to the presence of PIP3, see the contribution by Günther Gerisch in the same volume of this journal. We therefore decided to include a positive feedback loop in the model. It is approximated by a quadratic growth term in the equations for the bundled and the dendritic network, *k*_*b*3_*b*^2 ^and *k*_*d*3_*d*^2^. Inspired by the model introduced in Ref. [20], we furthermore introduce cubic terms, *k*_*b*4_*b*^3 ^and *k*_*d*4_*d*^3^, to approximate the concentration limiting effect of steric hinderance, *i.e.*, the concentration of filamentous actin cannot grow without bound.

How do the bundled and the dendritic networks interact? We assume that both networks mutually hinder each others growth, *i.e.*, in the presence of bundled network, the growth of the dendritic network will be slowed down and vice versa. This interaction is taken into account by including negative terms of the form *k*_*b*5_*d *and *k*_*d*5_*b *into the equations for the bundled and the dendritic networks, respectively. Summing up these processes, we obtain the following cubic equations for the temporal change in the concentrations of the bundled and the dendritic networks,(1)(2)

By definition, all rate constants *k*_*bi*_, *k*_*di *_with *i *= 1, 2, ..., 5, are positive numbers. Their values are not known. In first approximation, we assume that the rate constants are similar for both network types, *k*_*bi *_≈ *k*_*di*_. Note that we work with dimensionless quantities *b *and *d *throughout this article. Stationary states in the system can be identified by considering the intersection points of the two nullklines, *∂*_*t*_*b *= 0 and *∂*_*t*_*d *= 0

In order to account for the experimental observations, we choose the rate constants *k*_*bi*_, *k*_*di *_such that the following conditions are fulfilled. First, we require that at least two stable states may occur (bistability). This can be only achieved for a non-monotonous shape of the nullclines, *i.e.*, the nullclines have local minima and maxima. We thus require that the equation(3)

has two real and positive roots. Second, we require that bistability crucially depends on treatment with LatA. In absence of LatA, high concentrations of F-actin (both dendritic and bundled) prevail. On the other hand, for high concentrations of LatA the actin cortex decomposes. Only low concentrations of both dendritic and bundled F-actin are present in the cell under such conditions. As cells recover from drug treatment, *i.e. *at intermediate levels of LatA, bistability becomes predominant. It was shown that in the regime of wave formation, cells still show reduced amounts of F-actin [27]. To incorporate the effect of LatA in the model, we note that LatA influences actin polymerization by reducing the concentration of available G-actin via a tight binding to the G-actin/profilin complex [28,29]. Let us assume a linear dependence of the polymerization rates *k*_*b*1_, *k*_*d*1 _on the G-actin concentration *g*, so that  with *j *= *b, d*. An increased concentration of LatA will result in a reduced G-actin concentration and will be thus reflected in the model by reduced polymerization rates *k*_*b*1_, *k*_*d*1_.

**Figure 3:**
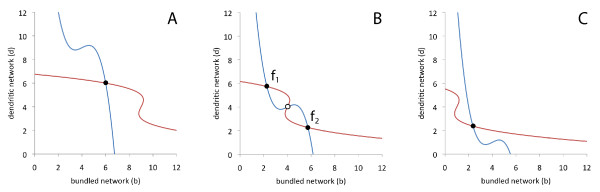
**Nullclines for the mono- and bistable regimes**. Nullclines in the (*b, d*)-phase plane for (A) high, (B) medium, and (C) low concentration of G-actin, corresponding to low, medium, and high levels of LatA, respectively. The stable fixed points are marked by filled circles, unstable fixed point by open circles. The G-actin concentrations are (A) *g *= 1, (B) *g *= 0.872, and (C) *g *= 0.795. The other parameters are , *k*_*j*2 _= 23.5, *k*_*j*3 _= 6, *k*_*j*4 _= 0.5 and *k*_*j*5 _= 1 with *j *= *b, d*.

In Figure 3, we display the two nullclines for different levels of LatA. In absence of LatA (*g *= 1), the system exhibits only one stable fixed point at high concentrations of bundled and dendritic actin (Figure 3A). As the concentration of LatA is inceased (corresponding to decreasing *g*), a bistable regime is found (Figure 3B). In this regime, one of the stable fixed points shows a high concentration of dendritic network and only low levels of bundled network (*f*_1_). The other stable fixed point is dominated by the bundled network with only low amounts of dendritic structures (*f*_2_). For high levels of LatA (low concentration of G-actin), the system eventually resumes monostable behavior with one stable steady state at low concentrations of both bundled and dendritic network (Figure 3C).

## 3. Discussion

We have proposed a simple model of actin dynamics that is based on the interplay of two structurally different states of F-actin, a bundled and a dendritic network. In the appropriate parameter regime, the model can account for bistable dynamics as is observed in TIRF microscopy recordings of *Dictyostelium *cells, preferentially during recovery from treatment with LatA. What are the conclusions and predictions that can be inferred from this model?

The two monostable regimes show stable steady states at high and low F-actin concentrations, respectively. They adequately reflect the cellular behavior in absence of LatA and at high LatA concentrations, see also Figure 3A und 3C. In both monostable regimes, the two network types are present in equal concentrations. In the bistable regime, the two stable fixed points are characterized by different network compositions. Although in both states the bundled and the dendritic structures coexist, the ratio of both network types is different, *i.e.*, each fixed point is clearly dominated by one of the two network types (the precise concentration values depend on the choice of the rate constants *k*_*bi*_, *k*_*di*_with *i *= 1, 2, ..., 5). At the fixed point *f*_1_, we encounter predominantly dendritic structures, whereas at the fixed point *f*_2_, the actin cortex is mostly composed of bundled network, see Figure 3B. This is in agreement with the experimentally observed distributions of actin associated proteins in the bistable regime. In one of the stable states, a high concentration of the Arp2/3 complex is observed (see Figure 2). Since Arp2/3 is characteristic for a dendritic network structure, we conjecture that this state corresponds to the fixed point *f*_1 _of our model. The other stable state is dominated by cortexillin and myosin II (see Figure 2). They are associated with anti-parallel bundles of F-actin. Thus, it seems reasonable to identify this state with the fixed point *f*_2 _in our model. The cell cortex can be considered as a spatially extended system, in which neighboring locations are coupled by diffusion of cytoskeletal components, by polymerization-induced spreading of f-actin structures, and possibly also by mechanical interactions. In such spatially extended bistable systems, perturbations may induce local transitions between the two fixed points that can spread in the form of a trigger wave through the system [24]. On the basis of the model presented above, we conjecture that the experimentally observed actin waves are trigger waves in a spatially extended bistable system.

**Figure 4:**
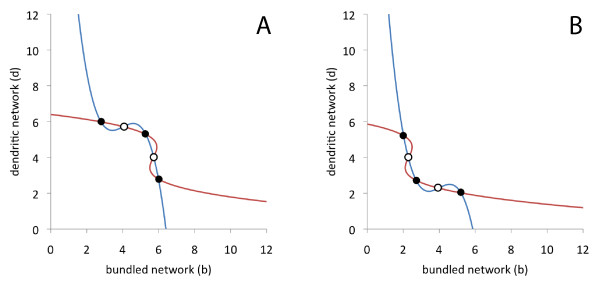
**Nullclines for multistable regimes**. Besides bistability, also multiple steady states may occur within narrow intervals of intermediate LatA levels. In (A) and (B), the nullclines of two such cases are displayed in the (*b, d*)-phase plane. The stable fixed points are marked by filled circles, unstable fixed point by open circles. The G-actin concentrations are (A) *g *= 0.915, and (B) *g *= 0.828. For the other parameters see the caption of Figure 3.

At the transition from monostable to bistable dynamics, our model predicts further multistable regimes. They strongly depend on the choice of parameters, as a change in the shape of the nullclines may alter the number of intersection point. Two examples are displayed in Figure 4. In both cases, three stable and two unstable fixed points are observed. The network composition in these stable states is similar to the compositions of the fixed points in the bistable and the adjacent monostable regimes. Such multistable states have not been reported in experiments. We hypothesize that the range of LatA concentrations, where such behavior may occur is very narrow and therefore rare to observe in experiments. However, our model predicts that multistable states of this type should also exist in the experimental system. Although not the object of the present study, we point out that also other dynamical regimes (*e.g. *oscillations or excitable behavior) may be observed in this model for different choices of the parameters.

The present model is purely phenomenological. It is intended as a minimal model that captures the essential dynamical features observed by TIRF microscopy in the cortex of *Dictyostelium discoideum *cells. Similar phenomenological approaches have been explored earlier to explain other experimental observations. In Ref. [20], a FitzHugh-Nagumo type system of equations was proposed to account for the transition from spots to waves in actin pattern formation. Motivated by the work of Ref. [20], we have included a cubic term in our model equation for F-actin. This term limits the concentration of filamentous actin and can be considered as a model for the effect of steric hinderance. Our modeling approach is thus closely related to the description proposed in Ref. [20]. In contrast to this earlier work, we do not explicitly include an actin degrading species as a dynamical variable in our model. Instead, we have conjectured that the composition of the actin cytoskeleton can be approximated by two distinct network structures that mutually hinder each others growth. The most basic growth and decay kinetics were assumed and rate constants were freely chosen. We note that the discussion of our model has been exclusively focused on temporal dynamics. No spatially extended system has been considered. A rigorously developed spatially extended version of the current model remains the subject of future work.

Bistability is encountered in many open, non-equilibrium systems, see *e.g. *[24] and references therein. It has been considered as the underlying dynamics of actin-driven transitions from nonpolarized to polarized cells [30] and was recently identified as one of the dynamical regimes in a polymer brush model that describes polymerization-driven force generation in the actin system [31]. Bistable mechanisms have also been introduced to explain directional responses of chemotactic cells [32,33]. In Ref. [34], we have proposed a bistable model for directional sensing that combines a common local excitation/global inhibition (LEGI) module with an additional bistable species. The biochemical nature of this bistable component was not specified. To exemplify the model, a general Michaelis-Menten-type rate law was assumed that led to a bistable kinetics. In the light of the current experimental evidence, we may conjecture that the actin system plays the role of the bistable agent that amplifies a shallow gradient stimulus into a switch-like directional response.

## 4. Conclusions

We have presented a phenomenological model to explain the formation of actin waves on the basis of a bistable mechanism. The model approximates the actin cytoskeleton as a structure that is composed of two distinct network types, a dendritic and a bundled network. From our model we draw the following conclusions. (a) Actin waves are trigger waves in a bistable system. (b) Actin waves separate two structurally distinct state of the actin system, the stable fixed points *f*_1 _and *f*_2_. (c) One of the two stable states is dominated by the dendritic, the other one by the bundled network. (e) At the transition from monostable to bistable behavior, more complex multistable regimes may occur. (f) We conjecture that bistability in the actin system plays a role for switch-like responses during directional sensing.
